# New Polyhedral Oligomeric Silsesquioxanes-Based Fluorescent Ionic Liquids: Synthesis, Self-Assembly and Application in Sensors for Detecting Nitroaromatic Explosives

**DOI:** 10.3390/polym10080917

**Published:** 2018-08-15

**Authors:** Wensi Li, Dengxu Wang, Dongdong Han, Ruixue Sun, Jie Zhang, Shengyu Feng

**Affiliations:** 1 Key Laboratory of Special Functional Aggregated Materials Ministry of Education, School of Chemistry and Chemical Engineering, Shandong University, Jinan 250100, China; anterlina@163.com (W.L.); dxwang@sdu.edu.cn (D.W.); handd1223@163.com (D.H.); sunruixue940622@163.com (R.S.); 2 National Engineering Technology Research Centre for Colloidal Materials, Shandong University, Jinan 250100, China

**Keywords:** fluorescent sensors, nitroaromatic explosives, polyhedral oligomeric silsesquioxane-based ionic liquids, self-assembly behaviors, thiol-ene ‘click’ reaction

## Abstract

In this paper, two different models of hybrid ionic liquids (ILs) based on polyhedral oligomeric silsesquioxanes (POSSs) have been prepared. Additionally, these ILs based on POSSs (ILs-POSSs) exhibited excellent thermal stabilities and low glass transition temperatures. ^1^H, ^13^C, and ^29^Si nuclear magnetic resonance (NMR) spectroscopy, Fourier transform infrared spectroscopy (FT-IR) and X-ray diffraction (XRD) were used to confirm the structures of the IL-POSSs. Furthermore, the spherical vesicle structures of two IL-POSSs were observed and were caused by self-assembly behaviors. In addition, we found it very meaningful that these two ILs showed lower detection limits of 2.57 × 10^−6^ and 3.98 × 10^−6^ mol/L for detecting picric acid (PA). Moreover, the experimental data revealed that the products have high sensitivity for detecting a series of nitroaromatic compounds—including 4-nitrophenol, 2,4-dinitrophenol, and PA—and relatively comprehensive explosive detection in all of the tests of IL-POSSs with nitroaromatic compounds thus far. Additionally, the data indicate that these two new ILs have great potential for the detection of explosives. Therefore, our work may provide new materials including ILs as fluorescent sensors in detecting nitroaromatic explosives.

## 1. Introduction

Ionic liquids (ILs), which have been recognized as a novel class of synthetic materials, have melting points below 100 °C [1,2,3]. With increasing demand for advanced materials, the development of novel ILs has been increasingly explored due to their unique properties [4], which include high ionic conductivity [5], high thermal stability, non-flammability, negligible vapor pressure, and a wide electrochemical stability window [6,7]. Due to the above advantages, ILs have been applied as functional materials in many chemical and industrial fields including fuel cells and lithium batteries [8], particular as catalysis solvents, capacitors, and electrochemical sensors [3]. Over the past few decades, polyhedral oligomeric silsesquioxane (POSS) materials have attracted much attention due to their unique thermal, chemical, and mechanical stabilities derived from their siloxane (Si–O–Si) frameworks [7,9], which have high bond energies. Furthermore, POSSs exhibit marvellous compatibility [10] with organic materials because of their inorganic core with various functional organic substituents [7,11,12]. Due to their many excellent merits, POSS materials have been extensively used in many fields, including as drug delivery agents [13], liquid crystalline materials [14,15], and light-emitting materials [16]. Given the aforementioned advantages of POSSs, introducing them into ILs is a reasonable approach to creating more novel and functional ILs.

The investigation of IL-POSS materials in different applications has been reported by many researchers. For example, Chujo and Tanaka et al. developed IL-POSSs with higher thermal stabilities than those of ILs linked to the side chains of POSSs because the IL-POSSs are comprised of inorganic frameworks with Si–O–Si bonds [6]. As such, these materials expand the range of applicable temperatures available to ILs. Tan [11] and his coworkers have also prepared IL-POSSs, but the synthetic route is not very convenient and the characterizations are not very comprehensive to illustrate the properties of IL-POSSs. In 2016, Li [17] and his coworkers have synthesized three IL-POSSs via the thiol-ene ‘click’ reaction, which is a very fast and efficient route to prepare different types of IL-POSSs for various purposes. The thiol-ene ‘click’ reaction allowed high yields, a variety of functional groups, easy purification, oxygen tolerance, etc. [18]. In addition, this approach has been demonstrated as an efficient and molecular modification procedure [19]. However, the fluorescent properties were not discussed, and there were no more applications of IL-POSSs as novel materials.

Due to the growing concerns about environmental protection and global terrorism activities, the demand for environmentally friendly sensor materials for the detection of nitroaromatic compounds has become a significant goal in the fields of materials chemistry and homeland security [20]. Therefore, fluorescent sensors [21] for the detection of nitroexplosives [22,23] are appealing to many researchers due to their simplicity, high sensitivity, high selectivity [24], cost-effectiveness [25], and fast response times.

Like trinitrotoluene (TNT) [26], picric acid (PA) is an extensively used explosive and is a dangerous environmental pollutant when present in groundwater, soil, etc. [27,28]. Thus, a safe and reliable approach to prevent environmental pollution and terroristic threats from PA is urgently needed. However, many sensors used to detect explosives present problems, such as a high cost [27,29] and difficult sample preparation. Therefore, there is an urgent need for better sensing materials for explosive detection.

IL-POSSs derived from imidazolium [27,30] have shown strong fluorescence and have been applied to the detection of PA in a previous report. However, compounds only functionalized with one or two imidazolium rings may be unable to show excellent fluorescence sensing abilities for detecting explosives compared to multifunctional compounds such as IL-POSSs. Based on this observation, we explored IL-POSS materials synthesized with more imidazolium rings in order to enhance their fluorescence sensitivities and then employed these IL-POSS materials for detecting a series of nitroaromatic compounds, including 4-nitrophenol, 2,4-dinitrophenol and PA. Interestingly, these IL-POSS materials demonstrated fast and efficient fluorescence quenching abilities for the detection of 4-nitrophenol, 2,4-dinitrophenol, and PA. The results presented here indicate that these IL-POSS materials, as new functional and environmentally friendly ionic liquids, could be used as fluorescent sensors in explosive detection.

## 2. Materials and Methods 

### 2.1. Materials

The starting materials (1-allylimidazole, 1-bromobutane) were purchased from commercial sources (Aladdin Co., Shanghai, China) and used without further purification. 2,2-Dimethoxy-2-phenylacetophenone (DMPA) was purchased from the Aladdin Co. (Beijing, China) and used as received. Tetrahydrofuran (THF) and toluene were purified according to a routine procedure and distilled over sodium before use. Octa(mercaptopropyl)silsesquioxane (denoted as POSS-SH) and octa(chloropropyl)silsesquioxane (denoted as POSS-Cl) were synthesized according to a method described in the literature [11,17].

### 2.2. Characterization and Measurements

The thiol-ene reaction mixture was irradiated with high-intensity UV light from a Spectroline model SB–100P/FA lamp (365 nm, 100 W, Spectroline Co., Westbury, NY, USA). ^1^H NMR, ^13^C NMR, and ^29^Si NMR spectra were recorded on a Bruker Avance-400 spectrometer (Bruker Co., Rheinstetten, Germany) using CDCl_3_ or a mixture of CD_3_OD and acetone-d_6_ as the solvent and without tetramethylsilane (TMS) as an internal reference. High resolution mass spectra (HRMS) spectra were obtained in the negative mode on an Agilent Technologies 6510 Q-TOF mass spectrometer (Agilent Co., Santa Clara, CA, USA). FT-IR spectra were recorded on a Bruker TENSOR-27 infrared spectrophotometer (Bruker Co., Ettlingen, Germany) via the KBr pellet technique within the wavenumber region from 4000 to 400 cm^−1^. X-ray diffraction (XRD) patterns were collected on a Bruker-D8 advanced X-ray diffractometer (Bruker Co., Karlsruhe, Germany) with a Cu radiation source (λ = 0.154 nm) operated at 40 kV and 30 mA using a Ni filter. Data were recorded in the range of 10° ≤ 2θ ≤ 80° at a scanning rate of 10 °/min. The luminescence (excitation and emission) spectra of the samples were recorded with a Hitachi F-4500 fluorescence spectrophotometer (Rigaku Co., Tokyo, Japan) equipped with a monochromatic Xe lamp as an excitation source. Thermal measurements were carried out using a TA Instruments SDTQ 600 (Mettler Co., Shanghai, China). The IL-POSSs were loaded into aluminium pans, which were then heated from −100 to 25 °C, cooled to −100 °C, and finally reheated to 25 °C. The heating and cooling temperature ramping rates were 10 °C/min. The DSC data are reported in this paper from the second heating cycle. TGA was performed using a Mettler Toledo TGA/DSC1 (Mettler Co., Shanghai, China) at a heating rate of 10 °C/min from room temperature to 700 °C under N_2_ (10 mL/min) at ambient pressure. To analyze the self-assembly behaviors of the IL-POSSs by TEM observation, samples were prepared by spreading a drop of aggregated solution onto a copper grid, followed by air drying at room temperature before testing on a JEM–1011 (100 kV) electron microscope (JEOL, Tokyo, Japan).

### 2.3. General Procedures to Synthesize the IL-POSSs

**Scheme 1:**
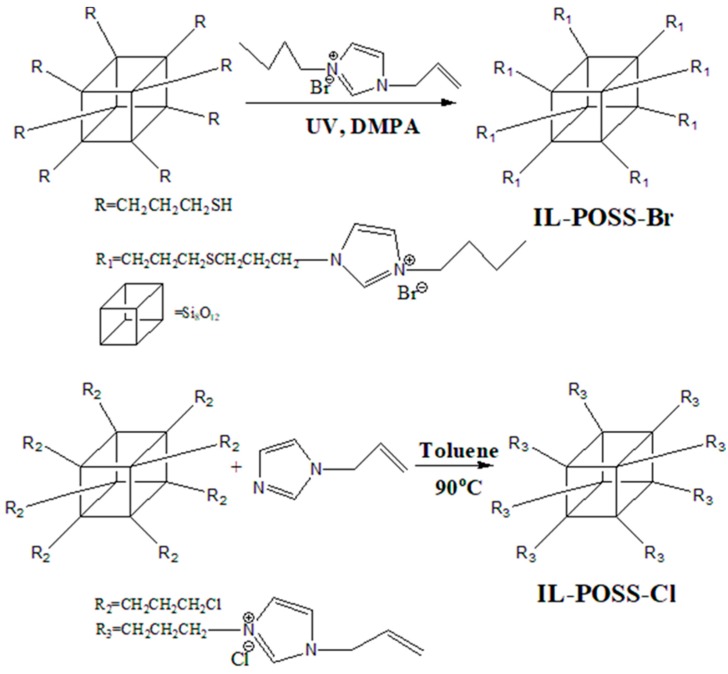
Synthesis of IL-POSS-Br and IL-POSS-Cl.

Allyl-min-Br was prepared through a quaternization reaction. IL-POSS-Br was synthesized via a classic procedure, as illustrated in Scheme 1. POSS-SH (1.06 g; 1 mmol), allyl-min-Br (1.96 g; 8 mmol), and DMPA (0.05 g; 2 wt %) were added to a transparent bottle with a 10 mL solvent mixture of CH_3_OH and CH_2_Cl_2_. The starting materials were then irradiated with a UV lamp for 15 min after dissolving completely. Finally, IL-POSS-Br was obtained after solvent evaporation at low pressure and vacuum drying at 60 °C for 24 h. IL-POSS-Cl was prepared through a simple but longer quaternization reaction. POSS-Cl (1.03 g; 1 mmol) and 1-allylimidazole (1.08 g, 10 mmol) were charged to a transparent bottle with 10 mL of toluene. The resultant mixture was heated to 85°C for at least 3 h, and the product was washed with a co-solvent of toluene and hexane several times. Finally, IL-POSS-Cl was obtained by vacuum drying at 60 °C for 24 h.

## 3. Results

### 3.1. Synthesis and Characterization

In this paper, we have synthesized two different IL-POSSs: IL-POSS-Br and IL-POSS-Cl. The synthetic routes are described in the following text:

First, to synthesize IL-POSS-Br, allyl-3-butylimidazolium bromide (allyl-min-Br) was prepared via the reaction of 1-allylimidazole with n-bromobutane. Afterwards, a thiol-ene reaction was carried out to synthesize IL-POSS-Br according to the route illustrated in Scheme 1 [17]. Fourier transform infrared (FT-IR) spectroscopy was used to characterize the structure and monitor the disappearance of the –SH groups (γ = 2565 cm^−1^) after 15 min of reaction, coinciding with the appearance of peaks assigned to the imidazole ring at 1580 and 1445 cm^−1^. Second, with respect to IL-POSS-Cl [11], the peaks corresponding to the imidazole rings were observed after the same amount of time. The peaks corresponding to the silsesquioxane frameworks of these two IL-POSS materials, such as Si–O–Si, appeared at 1050–1130 cm^−1^ (Figure S1). The ^1^H NMR spectra of the IL-POSSs are shown in Figures S2 and S3. For IL-POSS-Br, whereas the characteristic SH peak at 1.37 ppm and the CH=CH_2_ peak at approximately 5.5–6.0 ppm vanished, a new peak emerged at 2.25 ppm, and signals corresponding to the imidazole ring appeared at approximately 7.75 and 9.25 ppm. For IL-POSS-Cl, the characteristic imidazole ring peak and CH=CH_2_ peak are observed at approximately 7.70–9.25 ppm and 5.5–6.0 ppm, respectively. The ^13^C NMR spectra of IL-POSS-Br and IL-POSS-Cl are shown in Figure S4. These results demonstrate a complete reaction. Moreover, only one peak (δ = −66.8 ppm) was detected in the ^29^Si NMR spectrum of IL-POSS-Br (Figure S5) [31], illustrating that the POSS cage remains intact during the reaction [7]. Furthermore, we observed the appearance of two peaks at *m*/*z* = 749.1942 (IL-POSS-Br) and *m*/*z* = 948.2664 (IL-POSS-Cl) in the mass spectra (Figure S6), providing evidence that the targeted products were successfully prepared.

For further study, we employed X-ray diffraction (XRD) to estimate the structures of these two IL-POSSs (shown in Figure S7). Wide peaks appeared near 22.6° in the patterns of both IL-POSSs; such peaks are commonly observed in the XRD patterns of amorphous silica nanocomposites and are caused by the Si–O–Si bonds.

### 3.2. Thermal Properties

To demonstrate the thermal behaviors of the two IL-POSS materials, thermogravimetric analysis (TGA) and differential scanning calorimetry (DSC) were used to characterize the materials. IL-POSS-Br shows better thermal stability than IL-POSS-Cl, and the thermal decomposition temperatures (T_d_ at 5 wt % loss) of the materials are approximately 300 °C and 200 °C, respectively (Figure S8). Compared with the IL-POSS prepared by Tan and coworkers [11], the thermal stability of IL-POSS-Cl is slightly lower due to the CH=CH_2_ group. For IL-POSS-Br, some weight loss occurs at the beginning of the measurement, which is ascribed to the loss of volatile compounds. Consistent with the excellent structures of the IL-POSS materials, these results indicate that strong ionic interactions occur within them, enabling their application of over a wider range of fields.

DSC measurements were conducted under a nitrogen atmosphere and provided evidence that these two IL-POSSs both exhibit a single glass-transition temperature (*T*_g_). IL-POSS-Br exhibits an endotherm near −30 °C (Figure S9), whereas IL-POSS-Cl shows an endotherm near −20 °C (Figure S9). According to the data reported herein, we can deduce that the *T*_g_ was strongly affected by the anions and that, as expected, the presence of POSS substantially increased the *T*_g_ [32]. Obviously, the size of the anion strongly influenced the resultant *T*_g_, and the data suggest that the *T*_g_ decreases with increasing differences in the sizes of the cation and anion. In addition, only one glass transition is observed, which indicates that these anions were homogenously dispersed in the IL-POSSs.

### 3.3. Self-Assembly Behaviors

**Figure 1:**
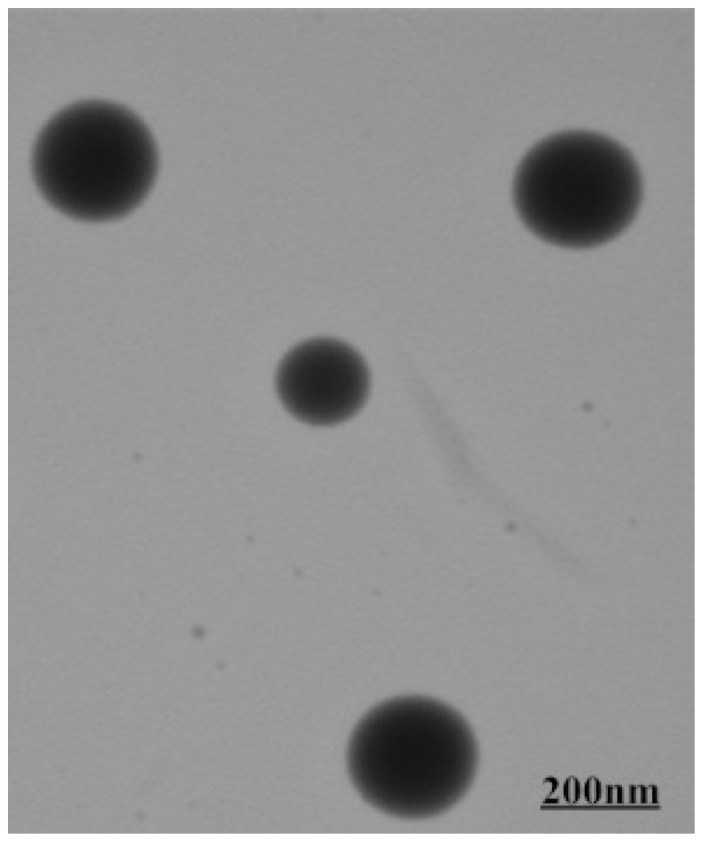
TEM image illustrating the self-assembly behaviors of IL-POSS-Br in ethanol.

**Scheme 2:**
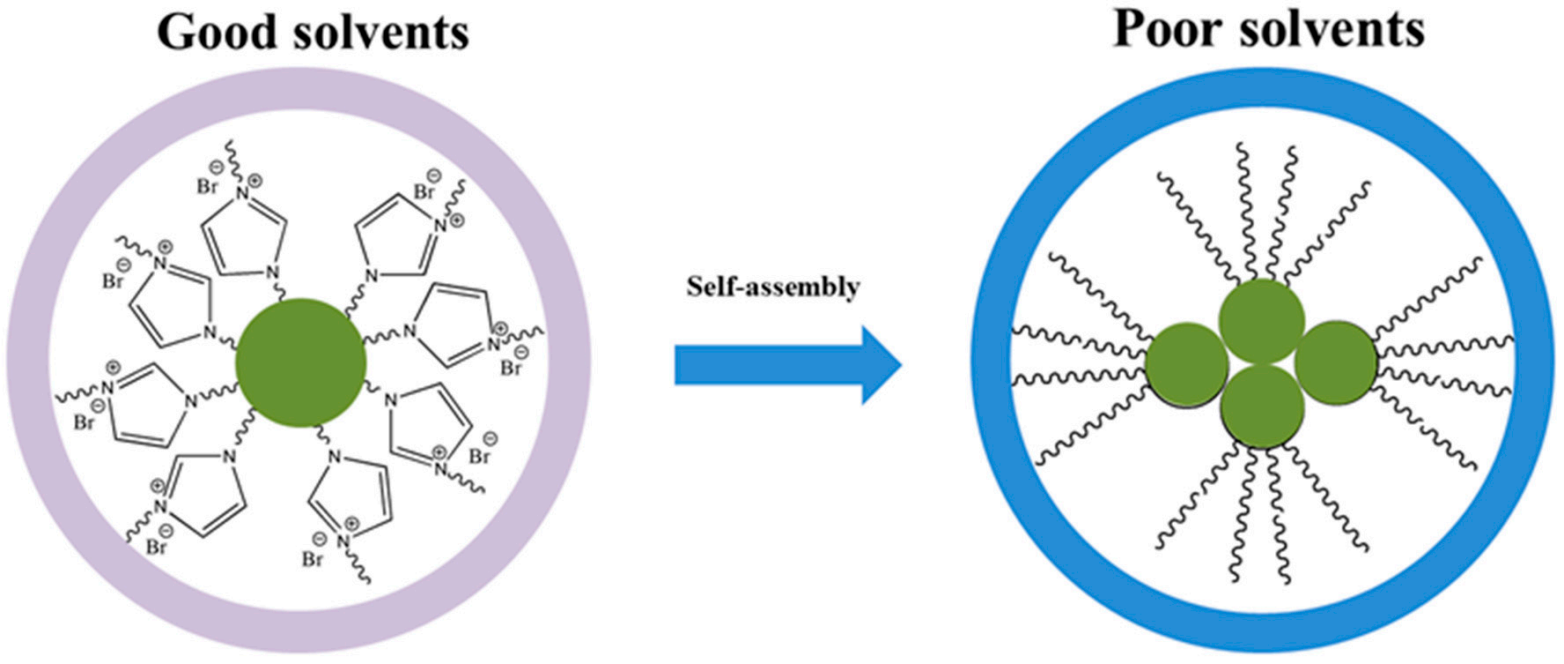
Proposed working mechanism for IL-POSS aggregation in ethanol. Reproduced with permission from [17]. Copyright Wiley 2016.

We also examined the self-assembly behaviors of the two IL-POSSs via transmission electron microscopy (TEM) to investigate their amphiphilic nature [33]. Using IL-POSS-Br as an example, our study shows that the average diameter of these spherical vesicles is approximately 200 nm when solvated in C_2_H_5_OH (shown in Figure 1). To better illustrate these self-assembly behaviors, we suggest a mechanism for vesicle formation (shown in Scheme 2) [17]. The combination of POSS-SH and imidazolium-containing side chains generated a dendritic ionic liquid supported by a POSS cage. These dendritic IL-POSSs can move freely when dissolved in good solvents. Nevertheless, in specific solvents, self-assembly occurred and spherical vesicles formed with POSS cages on the inside and anions on the outside. Obviously, strong electrostatic interactions are present in IL-POSSs [34,35], which causes the cations and anions to aggregate. These data suggest that the spherical vesicle shape is the lowest energy state and is driven by electrostatic interactions. These data suggest that the spherical vesicle shape is the lowest energy state driven by electrostatic interaction [6,36].

With respect to interfacial chemistry, we studied the static contact angle (CA) values of IL-POSS-Br and IL-POSS-Cl with distilled water as the test liquid. The CA of IL-POSS-Br was 69.8°, and the CA of IL-POSS-Cl was 66.1° (Figure S10). These results suggest that anions such as miscible halides strongly influence the miscibility of IL-POSSs and distilled water. In addition, CMC data (Figure S11) are 2.7 and 8.5 mM for IL-POSS-Cl and IL-POSS-Br, respectively. Based on this report, we can predict that ILs can be effectively integrated with amphiphilic molecules for two purposes: to introduce the excellent properties of ILs to the traditional self-assembly and aggregation of amphiphilic molecules and to further expand the development and application of ILs.

### 3.4. Optical Properties

**Figure 2:**
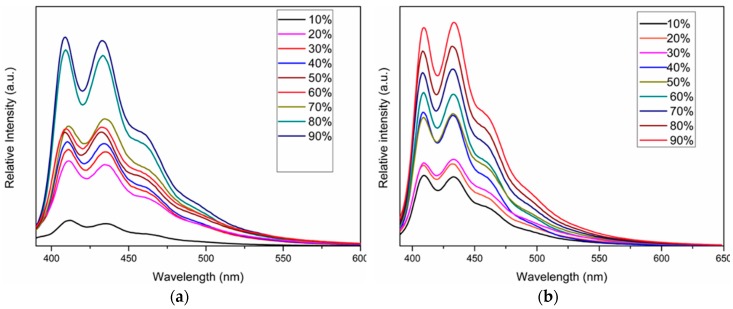
Emission spectra of IL-POSS-Br (**a**) and IL-POSS-Cl (**b**) (1 × 10^−6^ M).

For further investigation, the fluorescent properties of the IL-POSS materials were characterized. Both IL-POSSs exhibited two emission bands centred at approximately 410 nm and 430 nm when excited at 365 nm, as shown in Figure 2a,b. We propose two causes for the fluorescence of IL-POSSs: the fluorescent properties of the imidazole rings linked to the IL-POSSs and the fluorescence of POSS linked with mercaptopropyl. The splitting of the 3*d* orbital of the Si atom is caused by Si→S coordination bonds [37,38]; therefore, a *d*–*d* transition occurred due to the rearrangement of electrons in the split orbitals [31], and this transition is beneficial for the fluorescence of the POSS cages. In addition, the emission intensity decreased with decreasing concentration, consistent with the reported data for most organic luminescent compounds.

## 4. Discussion

### 4.1. IL-POSSs in the Detection of Nitroaromatic Explosives

**Figure 3:**
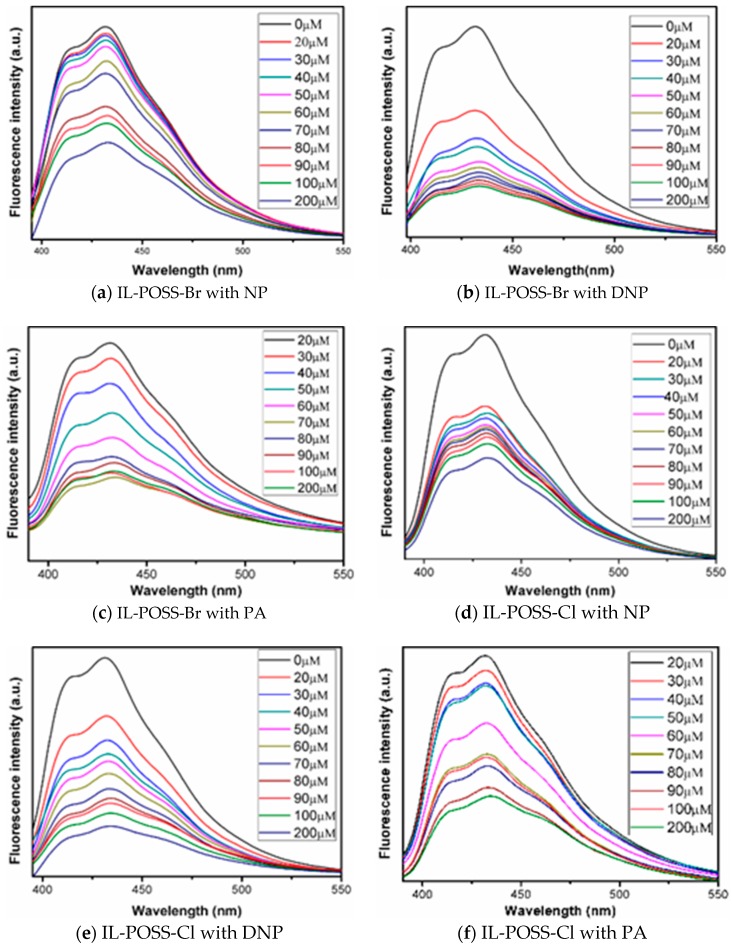
Emission spectra (**a**–**c**) belong to IL-POSS-Br upon the addition of 4-nitrophenol (NP), 2,4-dinitrophenol (DNP) and picric acid (PA) ethanol solution, respectively. Emission spectra (**d**–**f**) belong to IL-POSS-Cl upon the addition of NP, DNP, and PA, respectively.

**Figure 4:**
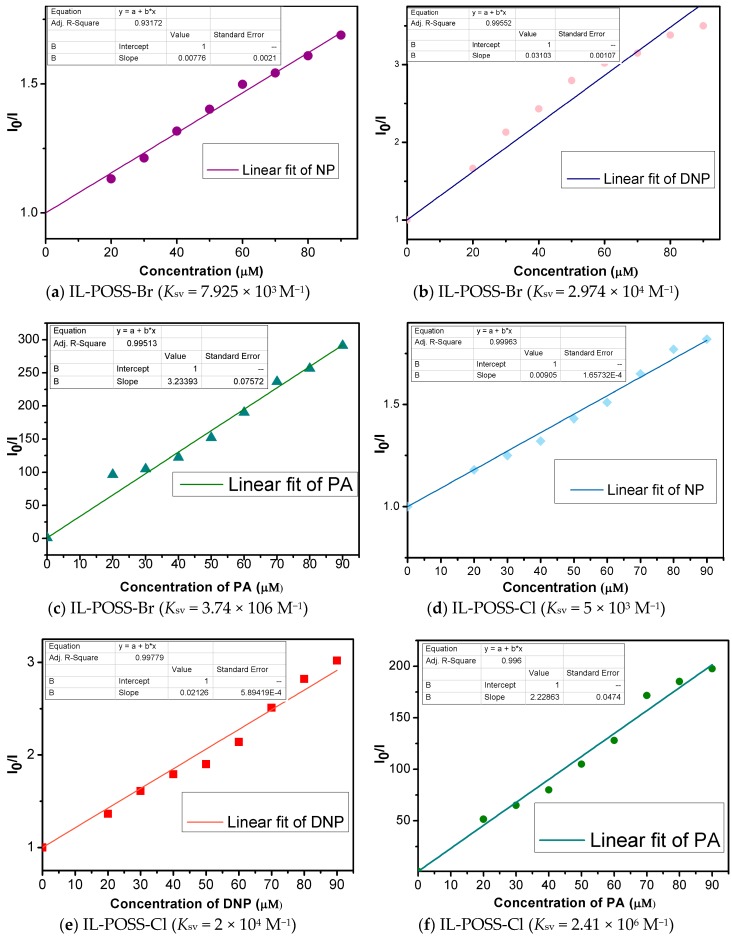
The Stern–Volmer curves (**a**–**c**) belong to IL-POSS-Br upon the addition of 4-nitrophenol, 2,4-dinitrophenol and picric acid ethanol solution, respectively. Emission spectra (**d**–**f**) belong to IL-POSS-Cl upon the addition of 4-nitrophenol, 2,4-dinitrophenol, and picric acid ethanol solution, respectively.

Based on the aforementioned excellent fluorescence of IL-POSSs, we explored their application in the detection of 4-nitrophenol (NP), 2,4-dinitrophenol (DNP) and picric acid (PA) in ethanol [39]. To examine the sensing abilities and monitor the fluorescent response of IL-POSS-Br and IL-POSS-Cl towards the three aforementioned nitroaromatic compounds, the two IL-POSSs were dissolved in ethanol with gradually increasing NP, DNP, and PA contents, as shown in Figure 3. The spectra clearly show that substantial fluorescence quenching occurred in both IL-POSS materials. The fluorescence intensity gradually weakened with increasing concentrations of the explosives. The sensing studies were performed by recording the changes with excitation at 365 nm. To further investigate the sensitivity for the detection of NP, DNP, and PA, the Stern–Volmer plots were obtained. As shown in Figure 4, the respective Stern–Volmer plots of NP, DNP, and PA in the presence of IL-POSS-Br and IL-POSS-Cl better demonstrate the sensing abilities of the IL-POSSs. The Stern–Volmer plot is linear when the concentration of PA is low. According to the literature [29], the Stern–Volmer constants (K_sv_) for NP, DNP, and PA can be calculated from the slopes of the Stern–Volmer plots. In Figure 4a–c, the Stern–Volmer constants (*K*_sv_) of IL-POSS-Br are 7.925 × 10^3^, 2.974 × 10^4^, and 3.74 × 10^6^ M^−^^1^ for NP, DNP, and PA, respectively. In Figure 4d–f, the Stern–Volmer constants (*K*_sv_) of IL-POSS-Cl are 5 × 10^3^, 2.5 × 10^4^, and 2.41 × 10^6^ M^−1^ for NP, DNP, and PA, respectively. Therefore, the quenching efficiencies of both IL-POSSs in the presence of the studied explosives decrease in the order PA > DNP > NP, which is consistent with the fluorescence intensities observed in Figure 3.

To better illustrate the quenching behaviors of these materials, we calculated their limits of detection (LDs, LD = 3 × σ/K, σ = standard deviation of blank measurement = 3.20) from the slopes via the approximate linear relationship between the fluorescence intensity of the IL-POSS and the concentration of the relevant explosives; the results are shown in Figure 4. The LDs of IL-POSS-Br are 1.21 × 10^−3^, 3.23 × 10^−4^, and 2.57 × 10^−6^ mol/L for NP, DNP, and PA, respectively, and the LDs of IL-POSS-Cl are 1.92 × 10^−3^, 3.84 × 10^−4^, and 3.98 × 10^−6^ mol/L for NP, DNP, and PA, respectively.

**Figure 5:**
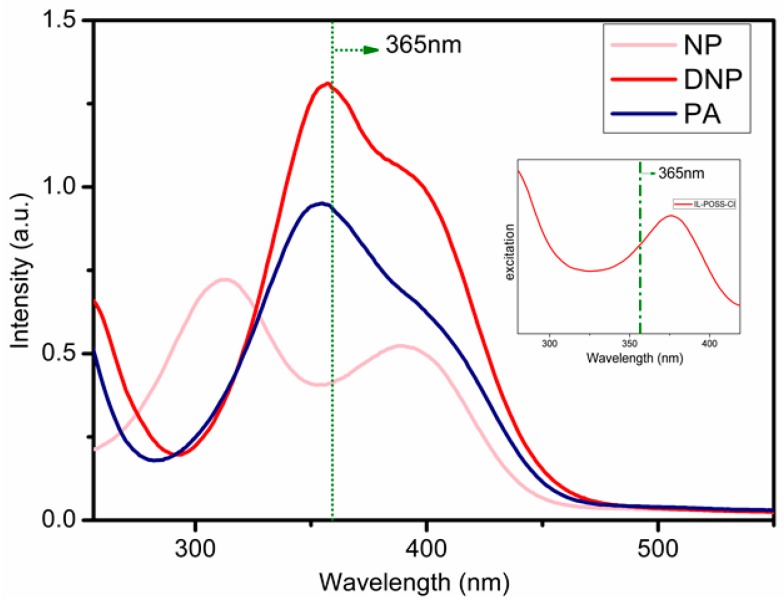
UV–vis absorption spectra of various analytes in ethanol and the excitation spectrum of IL-POSS-Cl in an ethanol suspension.

The aforementioned phenomenon is attributed to two factors [39]: (1) electron transfer from the imidazole rings of IL-POSSs to the electron-deficient nitroaromatic compounds, which enables electron transfer to occur among the functional groups; and (2) competitive absorption or the inner-filter effect (IFE) [40], which is caused by other absorbents or the simultaneous absorption of the excitation and emission lights of the fluorescent materials in both of the detection systems. In Figure 5, the absorption bands of all the explosives show obvious overlap with the excitation spectra of IL-POSS-Cl (the excitation wavelength at 365 nm is included). Thus, the absorption by the explosives filters the light absorbed by the IL-POSSs, resulting in fluorescence quenching. With regards to the fluorescence emission mechanism, after the fluorescent materials absorbed the light or energy, the electrons would be transitioned from the ground state (S_0_) to the excited states (S_1_, S_2_) [41], and due to the unstable state, they would release energy to achieve an absorption competition of the light source energy between the materials and the analytes after a series of vibrations. Consequently, the coexistence of the electron transfer effect and competitive absorption leads to fluorescence quenching behaviors. These data demonstrate that IL-POSS-Br and IL-POSS-Cl show high selectivity for the detection of NP, DNP, and PA.

### 4.2. Simple and Efficient Testing of IL-POSSs as Sensors for Detecting Nitroaromatic Explosives

**Figure 6:**
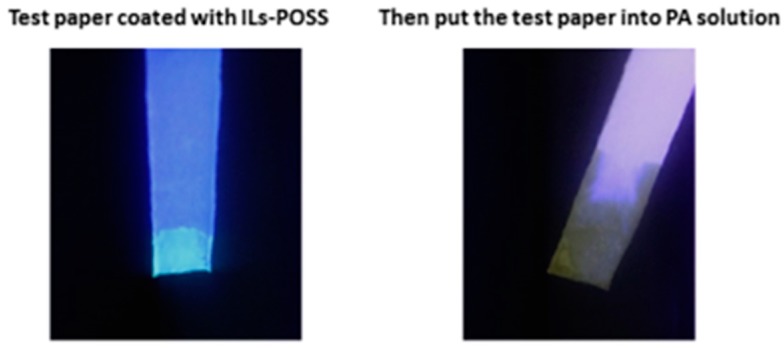
Photographs of test paper coated with IL-POSSs before and after being immersed into the aqueous solution of picric acid.

For a better visual observation, the IL-POSSs were tested as fluorescent sensors for the detection of PA (shown in Figure 6). Under a UV lamp (365 nm), test papers coated with IL-POSSs clearly changed colors after being immersed into an aqueous solution of PA [27]. Unlike other fluorescent sensors, IL-POSSs have two advantages: they are more environmentally friendly and more adaptable to ‘green’ chemistry than certain other sensors because of their basically green synthetic approach, and these IL-POSSs can be expanded to applications in aqueous systems. In comparison, traditional fluorescent sensors are often hydrophobic, which limits their application.

## 5. Conclusions

Overall, in this paper, two IL-POSS materials were successfully prepared in high yields via different methods. By comparison of the synthesis time, it has been demonstrated that the thiol-ene ‘click’ reaction is easily applied in the preparation of IL-POSSs due to its fast and efficient merits. Due to the POSS cage, both IL-POSSs exhibit good thermal stabilities and low glass-transition temperatures, which endows them with a wide range of applicable temperatures. In addition, the unique amphiphilic nature of the two IL-POSSs allows them to aggregate into spherical vesicle structures in select solvents. Furthermore, based on the fluorescent properties of IL-POSSs, we have identified another application, fluorescent sensors that can efficiently detect NP, DNP, and PA, and the comparison of the sensitivities has illustrated that IL-POSS-Br performed better than IL-POSS-Cl with regards to the detection of PA. Additionally, the simple test in Figure 6 is visual proof that the IL-POSSs can be employed as sensors in detecting PA, although this test provides only preliminary evidence. Finally, more functional IL-POSS materials can be fabricated through this reaction and used as effective fluorescent sensors in the future.

## Supplementary Materials

The following are available online at http://www.mdpi.com/2073-4360/10/8/917/s1, Figure S1: FTIR spectra of IL-POSS-Br and IL-POSS-Cl, Figure S2: ^1^H spectra of IL-POSS-Br and HS-POSS. Note: * represents solvent peaks, Figure S3: ^1^H spectra of IL-POSS-Cl. Note: * represents solvent peaks, Figure S4: ^13^C spectra of IL-POSS-Br and IL-POSS-Cl. Note: * represents solvent peaks, Figure S5: ^29^Si spectrum of IL-POSS-Br, Figure S6: HRMS spectra of IL-POSS-Br and IL-POSS-Cl, Figure S7: XRD spectra of IL-POSS-Br and IL-POSS-Cl, Figure S8: TGA curves of IL-POSS-Br and IL-POSS-Cl, Figure S9: DSC curves of IL-POSS-Br and IL-POSS-Cl, Figure S10: Contact angles (CA) of IL-POSS-Br and IL-POSS-Cl.
