# Supplementary material for: New Polyhedral Oligomeric Silsesquioxanes-Based Fluorescent Ionic Liquids: Synthesis, Self-Assembly and Application in Sensors for Detecting Nitroaromatic Explosives
Source: Polymers (Basel). 2018 Aug 15;10(8):917. doi: 10.3390/polym10080917 (PMC6404091; doi:10.3390/polym10080917)
Supplement: Supplementary file 1 [file polymers-10-00917-s001.pdf]

New polyhedral oligomeric silsesquioxanes-based fluorescent ionic liquids: synthesis, self-assembly and application in sensors for detecting nitroaromatic explosives

Wensi Li, Dengxu Wang, Dongdong Han, Ruixue Sun, Jie Zhang*, Shengyu Feng*,

Experimental Section

Materials.

Starting materials (1-allylimidazole, 1-bromobutane) were purchased from commercial resources and used without further purification. 2,2–Dimethoxy–2–phenylacetophenone (DMPA) was purchased from Aladdin Co. (China) and used as received. Tetrahydrofuran (THF) and toluene were purified according to routine procedure and distilled over sodium before use. Octa(mercaptopropyl)silsesquioxane (denoted as POSS-SH) and octa(chloropropyl) silsesquioxanes (denoted as POSS-Cl) was synthesized according to the literature.

Characterization and measurements

The thiol-ene reaction was irradiated by high-intensity UV on a Spectroline Model SB–100P/FA lamp (365 nm, 100 w). ^{1}H NMR, ^{13}C NMR, and ^{29}Si NMRB spectra was recorded on a Bruker Avance-400 spectrometer by using $CDCl_3$ or mixture of CD_3OD and Acetone-d_6 as solvent and without tetramethylsilane (TMS) as an internal reference. HRMS spectra were obtained through negative mode on Aglient Technologies 6510 Q-TOF mass spectra. Fourier transform infrared spectra (FT-IR) were measured on a Bruker TENSOR-27 infrared spectrophotometer with KBr pellet technique within the 4000-400 cm^{-1} region. X-ray diffraction spectra was collected on a Bruker-D8 Advanced X-ray diffractometer with Cu (λ=0.154nm) irradiation at 40kV and 30mA using a Ni filter. Data were recorded in the range of 2θ=10°-80° at the scanning rate of 10°/min. Luminescence (excitation and emission) spectra of the samples were determined with a Hitachi F-4500 fluorescence spectrophotometer using a monochromated Xe lamp as an excitation source. measurements were studied using SDTQ 600 of TA Instruments. The ILs-POSS were loaded in aluminum pans,

then heated from –100 to 25°C, cooled to –100°C, and finally reheated to 25°C. The heating and cooling temperature ramping rates were 10°C/min. The DSC data are reported in this paper from the second heating cycle. TGA was performed on a Mettler Toledo TGA/DSC1 with heating rate of 10°C/min from room temperature to 700°C under N_2 (10mL/min) at ambient pressure. Self–assembly behaviors of ILs-POSS for TEM observation were prepared by spreading a drop of aggregate solution on a copper grid followed by air drying at room temperature before the test on a JEM–1011 (100 kV) electron microscopy (JEOL, Japan).

General procedures to synthesize ILs-POSS

1-ally-3-butylimidazolium bromide (allyl-min-Br) was prepared through quaterisation reaction. IL-POSS-Br was synthesized via a classic procedure illustrated in the Scheme 1. POSS-SH (1.06g; 1mmol), Allyl-min-Br (1.96g; 8mmol), and DMPA (0.05g; 2wt%) were charged to a transparent bottle with a 10ml mixture solvent of CH_3OH and CH_2Cl_2. Then the starting materials were irradiated by UV lamp for 15 minutes after dissolved completely. Finally, IL-POSS-Br was obtained after solvent evaporation at low pressure and vacuum drying at 60°C for 24h. IL-POSS-Cl was prepared through simple but longer time reaction—quaterisation. POSS-Cl (1.03g; 1mmol) and 1-allylimidazole (1.08g, 10mmol) were charged to a transparent bottle with 10ml solvent of toluene. Then the mixture was heated to 85°C for 3h at least and the product was washed with a co-solvent of toluene and hexane several times. In the end, IL-POSS-Cl was obtained by vacuum drying at 60°C for 24h.

Data of IL-POSS-Br:

^{1}H NMR (400 MHz, MeOD) δ 9.23, 7.75, 4.42, 3.96, 2.98, 2.62, 1.92, 1.56, 1.16, 0.81

^{13}C NMR (101 MHz, MeOD) δ 136.07, 122.59, 51.52, 49.39, 47.96, 47.32, 33.97, 27.44, 19.16, 12.62 , 10.62.

^{29}Si NMR (79 MHz, MeOD) δ -66.71. Elemental analysis: Calc. N 7.53, C 41.94, H 6.45, S 8.60; Found N 7.49, C 40.83, H 6.43, S 8.573%. Yield: 90%

HRMS: 749.1942

Data of IL-POSS-Cl:

^{1}H NMR (400 MHz, $CDCl_3$) δ 7.70, 7.26, 6.99,6.12, 5.61, 5.58,5.31, 5.21, 4.60, 2.01,

1.84,0.81.

^{13}C NMR (101 MHz, MeOD) δ 137.04, 133.48, 130.83, 127.61, 122.63, 122.46, 120.65, 119.54, 117.18, 51.50, 48.93, 48.10, 47.89, 47.68, 47.04.

^{29}Si NMR (79 MHz, MeOD) δ -67.07. Elemental analysis: Calc. N 11.79, C 45.47, H 5.89; Found N 11.63, C 45.16, H 5.452%. Yield: 87%

HRMS: 978.2664

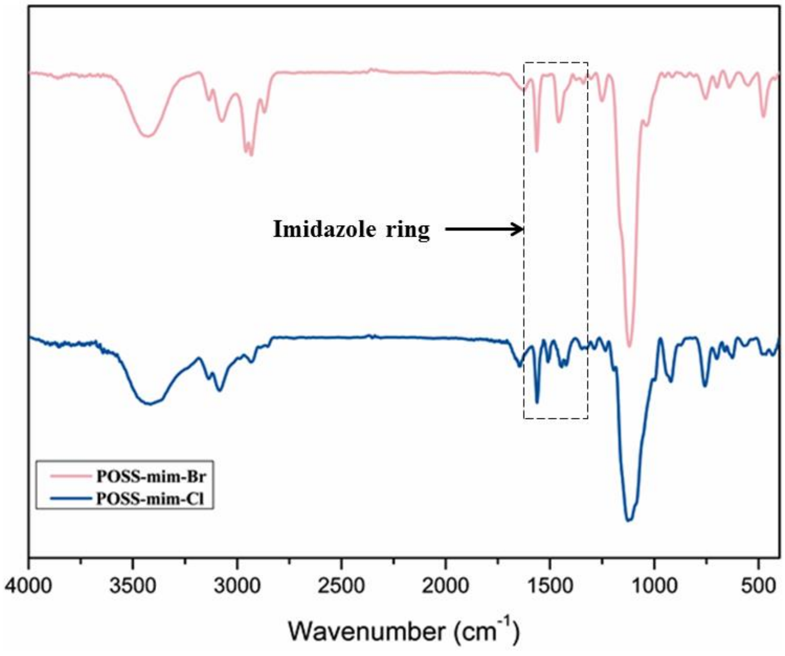

Figure S1 FTIR spectra of IL-POSS-Br and IL-POSS-Cl

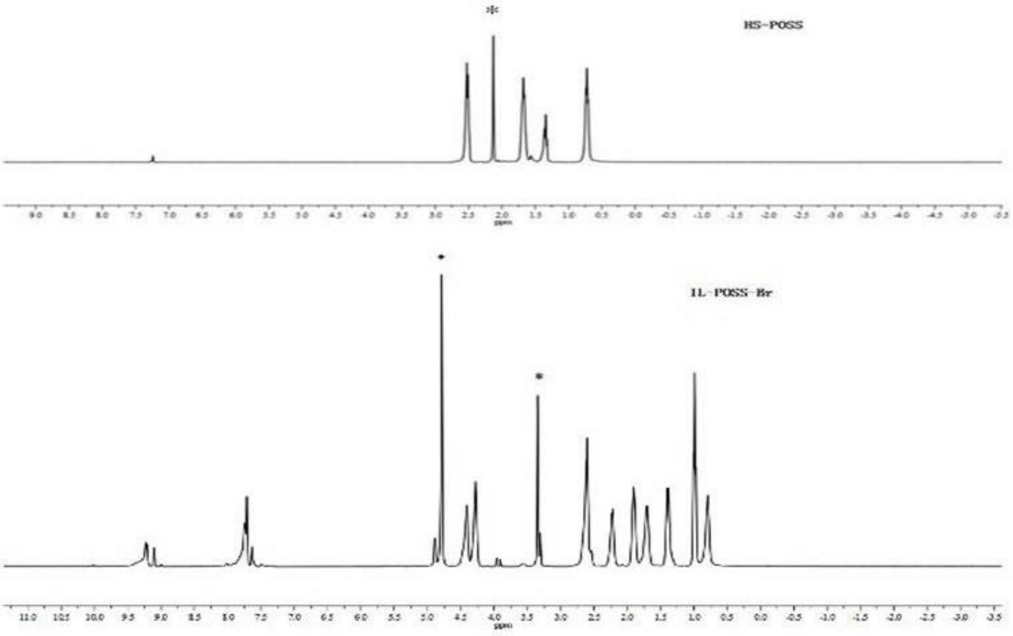

Figure S2 ^{1}H spectra of IL-POSS-Br and HS-POSS. Note: * represents solvent peaks.

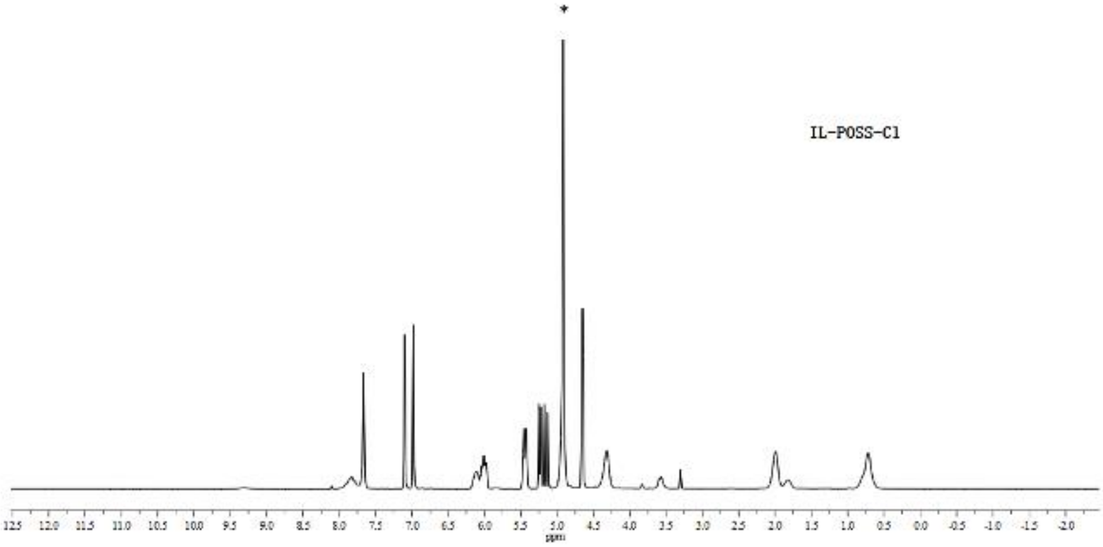

Figure S3 ^{1}H spectra of IL-POSS-Cl. Note: * represents solvent peaks.

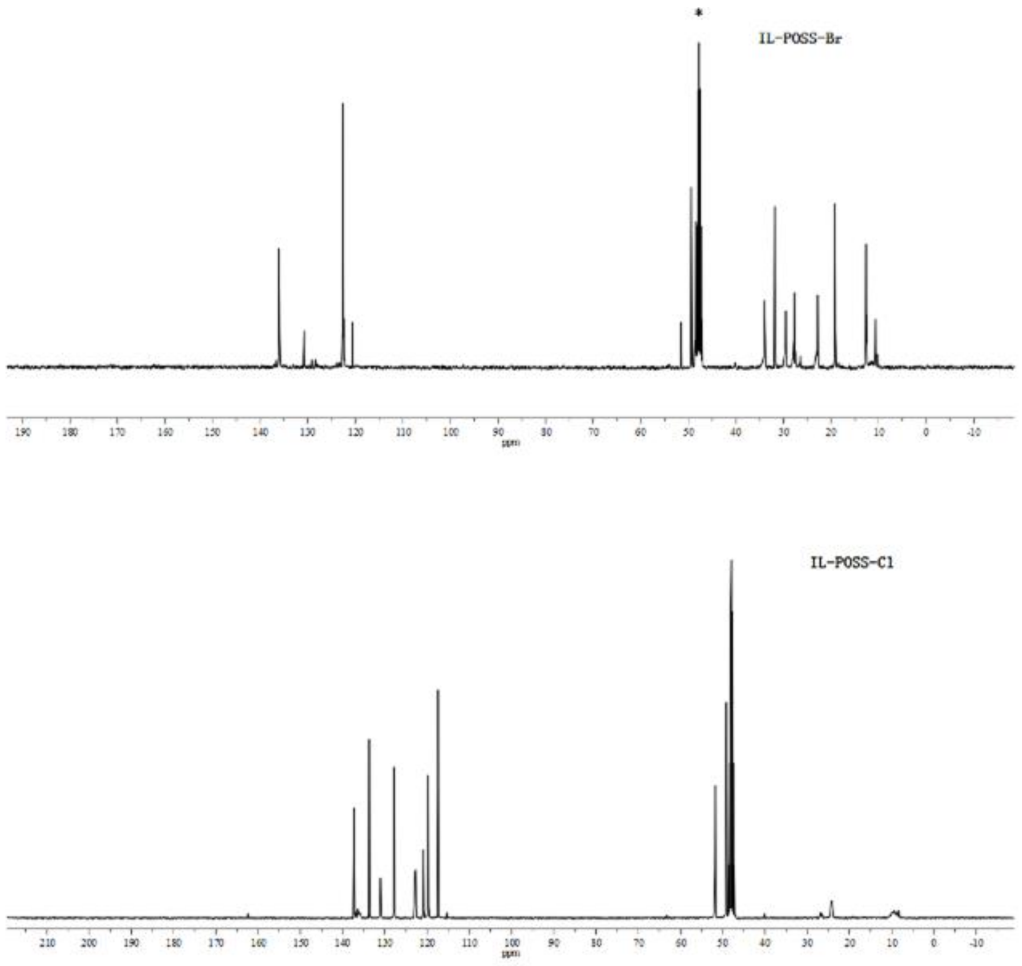

Figure S4 ^{13}C spectra of IL-POSS-Br and IL-POSS-Cl. Note: * represents solvent peaks.

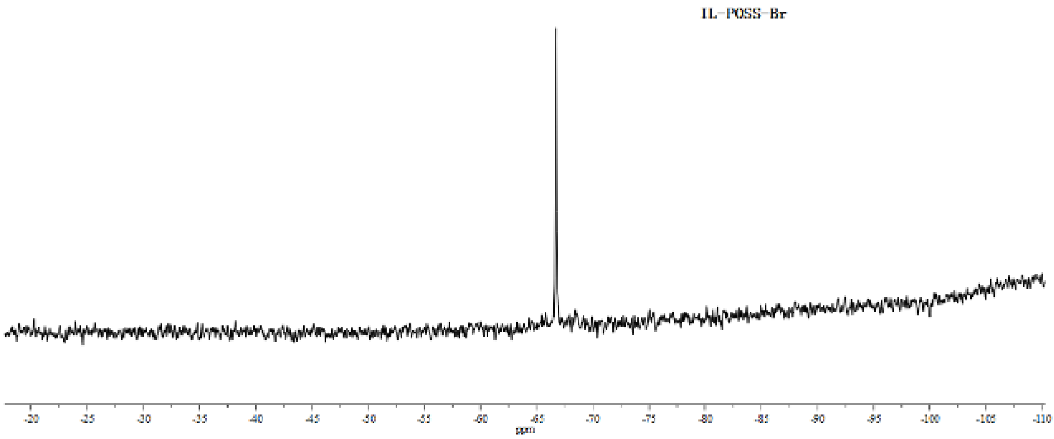

Figure S5 ^{29}Si spectrum of IL-POSS-Br

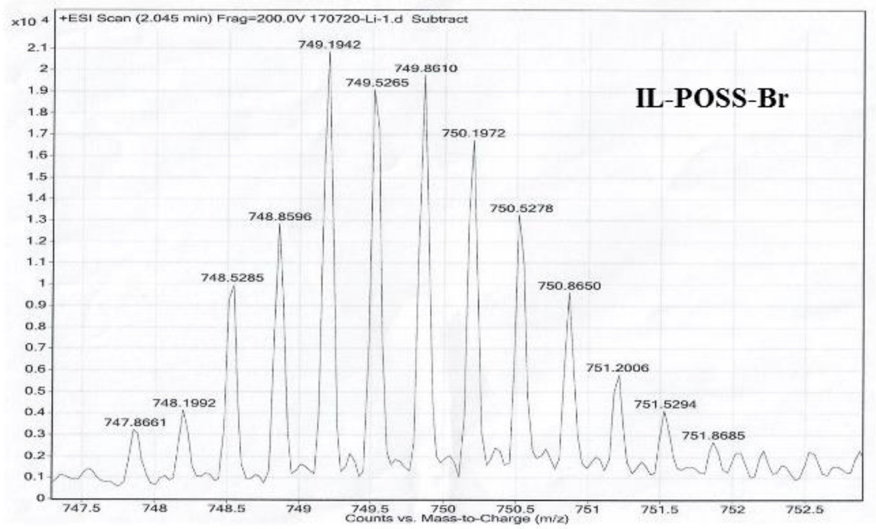

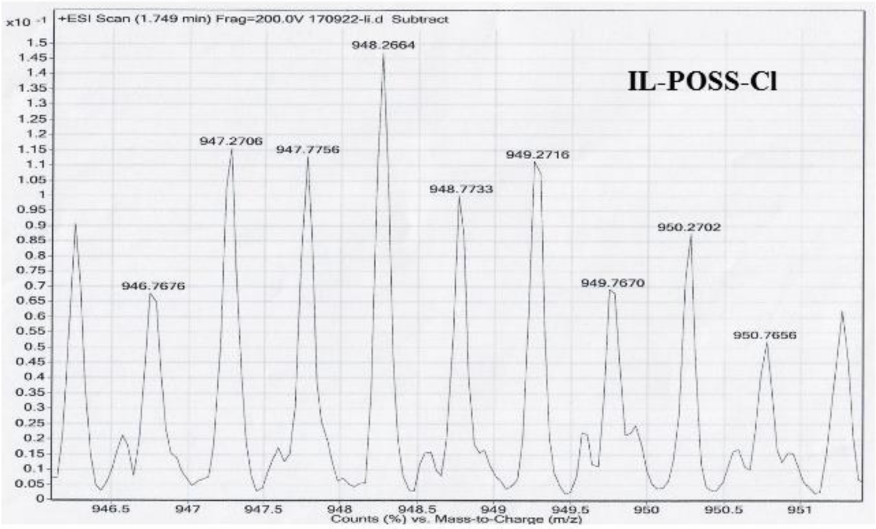

Figure S6 HRMS spectra of IL-POSS-Br and IL-POSS-Cl.

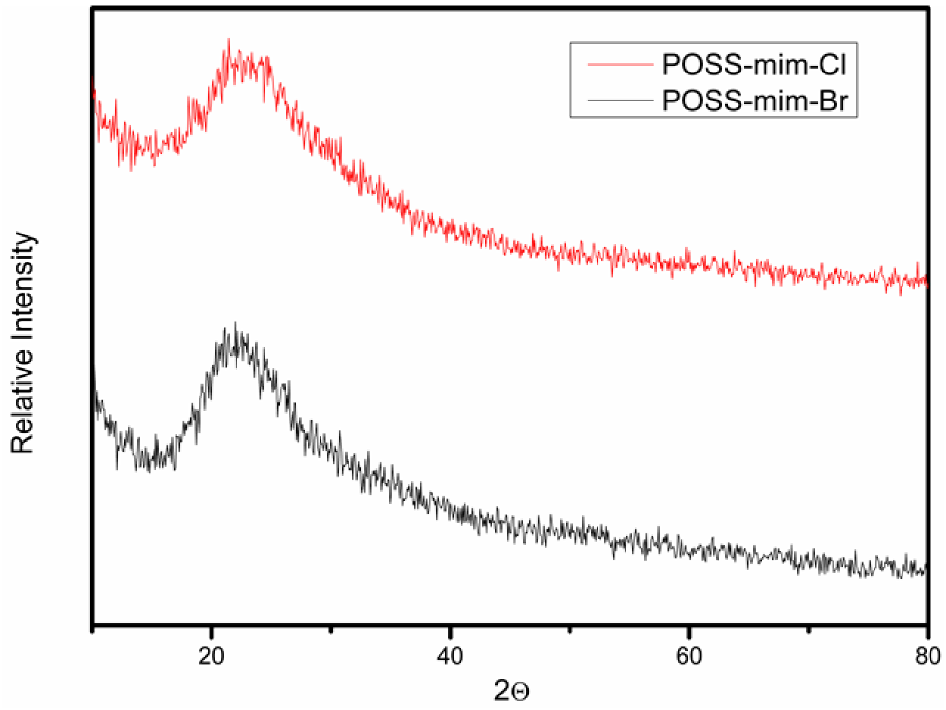

Figure S7 XRD spectra of IL-POSS-Br and IL-POSS-Cl.

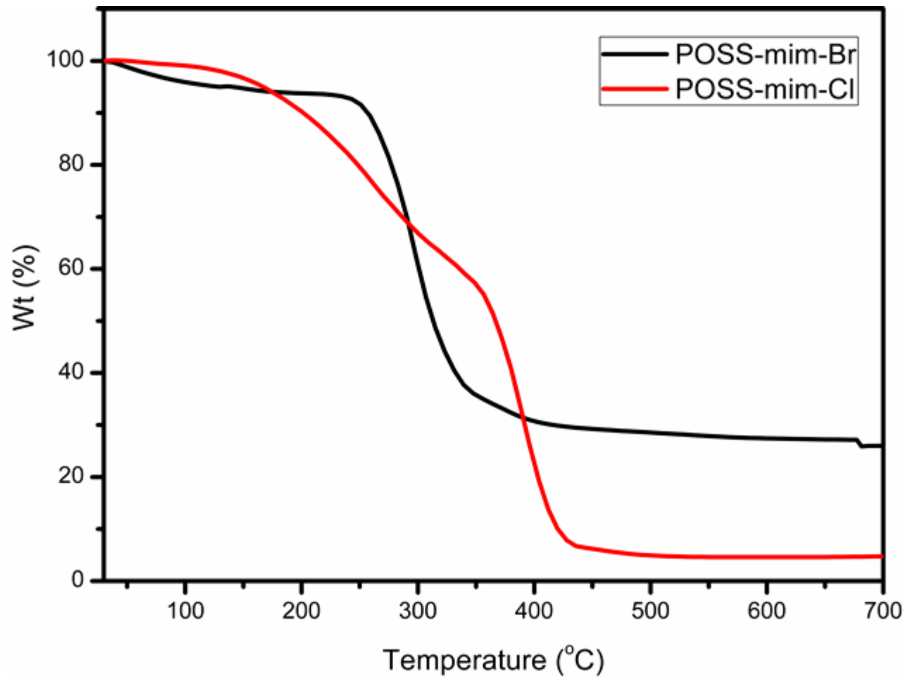

Figure S8 TGA curves of IL-POSS-Br and IL-POSS-Cl.

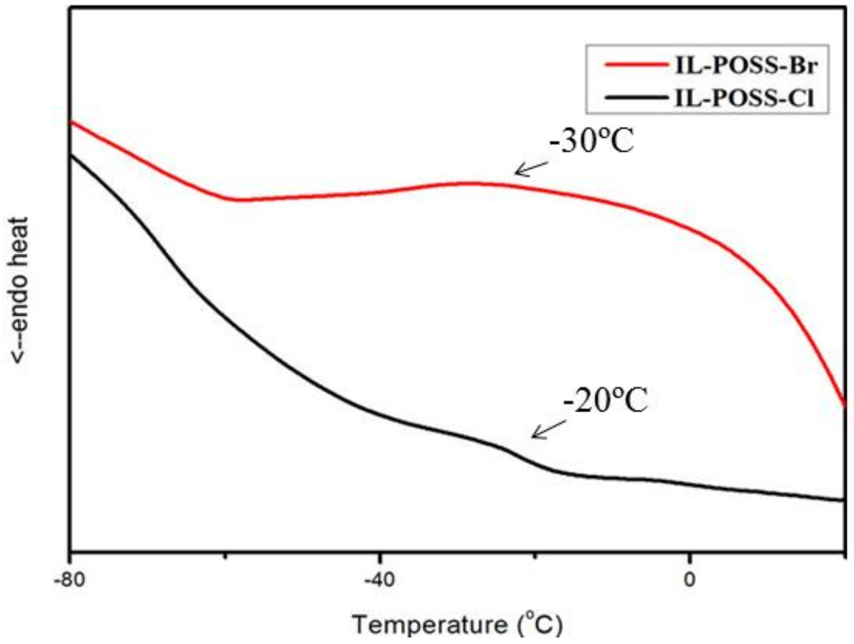

Figure S9 DSC curves of IL-POSS-Br and IL-POSS-Cl.

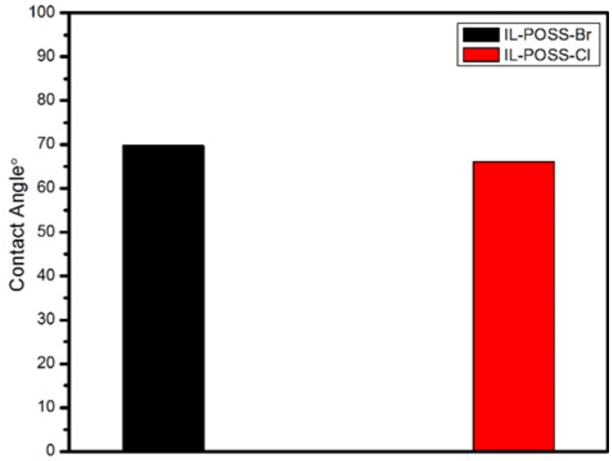

Figure S10. Contact angles (CA) of IL-POSS-Br and IL-POSS-Cl.

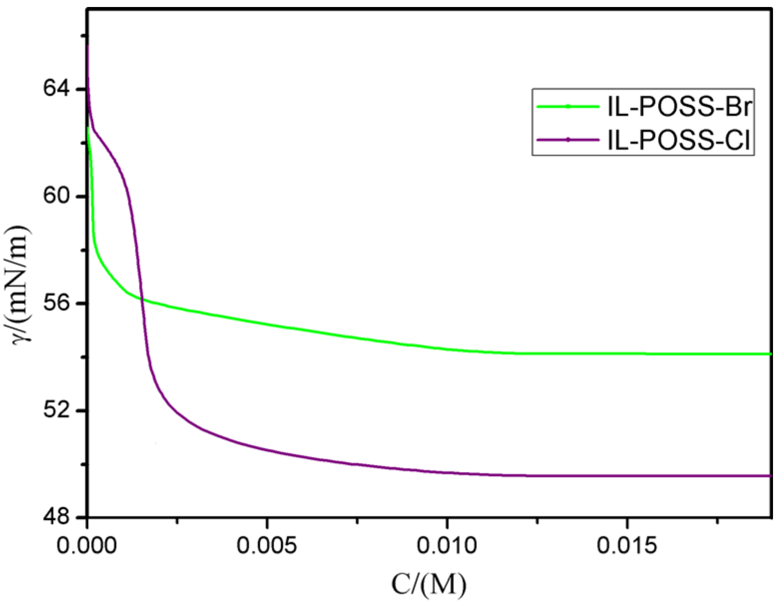

Figure S10. Surface tension of IL-POSS-Br and IL-POSS-Cl in aqueous solutions as a function of their concentrations at 25ºC.
